# Electronic Structure of Fullerene Nanoribbons

**DOI:** 10.1021/acsnano.5c08991

**Published:** 2025-07-30

**Authors:** Bo Peng, Michele Pizzochero

**Affiliations:** † Theory of Condensed Matter Group, Cavendish Laboratory, 2152 University of Cambridge, Cambridge CB3 0HE, United Kingdom; ‡ Department of Physics, 1555 University of Bath, Bath BA2 7AY, United Kingdom; § School of Engineering and Applied Sciences, Harvard University, Cambridge, Massachusetts 02138, United States

**Keywords:** nanoribbons, monolayer fullerene networks, first-principles, edge states, density functional theory

## Abstract

Using first-principles calculations, we examine the electronic structure of quasi-one-dimensional fullerene nanoribbons derived from two-dimensional fullerene networks. Depending on the edge geometry and width, these nanoribbons exhibit a rich variety of properties beyond conventional quantum confinement, including direct and indirect band gaps, positive and negative effective masses, edge and bulk states, as well as dispersive and flat bands. Our findings establish a comprehensive understanding of the electronic properties of fullerene nanoribbons, with potential implications for the design of future nanoscale devices.

## Graphical abstract

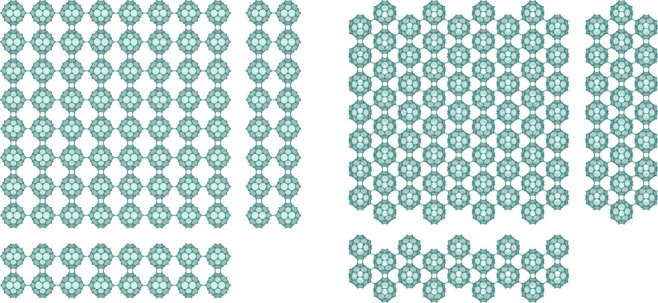

The edges of graphene exhibit intriguing physical properties, which have motivated the fabrication of graphene nanoribbons—a class of nanoscale materials composed of quasi-one-dimensional strips of hexagonally bonded carbon atoms. The exploration of graphene nanoribbons has opened new avenues for both fundamental research^1,2^ and future technology.^3,4^ Their structural and electronic properties can be controlled through width and edge geometry,^5^ serving as new degrees of freedom to achieve target structures and functionalities, e.g., heterojunctions with tunable band gaps.^6–8^ Additionally, the electronic properties of graphene nanoribbons can be controlled via chemical or electric approaches, leading to rich electronic phases, including Dirac semimetallic,^9,10^ half-metallic,^11,12^ magnetic,^13,14^ and topological^15–17^ phases. Because carbon can form rich allotropes, the search for carbon nanoribbons beyond graphene offers new avenues for tailored physical and chemical properties.^18,19^

The family of carbon-based two-dimensional materials has recently expanded with the introduction of monolayer fullerene (C_60_) networks,^20^ which offer a promising platform to realize potential applications in photocatalysis,^21–26^ thermal devices,^27–29^ nanofiltration,^30–32^ and photodetectors,^33–35^ within an ultrathin (<1 nm) molecular nanostructure. Yet, a thorough investigation of the properties on the edges of such monolayers is missing, with earlier studies being restricted only to polymeric C_60_ chain with a width of a single molecule.^36–45^ Understanding the impact of edges on monolayer fullerene networks is an issue of particular relevance to experiments, given that many structural phases of these networks tend to split into nanoribbons with increasing temperature or under mechanical strain.^46–48^

Here, we employ first-principles calculations to investigate the structural and electronic properties of fullerene nanoribbons derived from the experimentally known structural phases of monolayer fullerene networks. Depending on the edge geometry and width, we show that a variety of electronic properties, e.g., direct/indirect band gaps and negative/positive carrier effective masses, can be obtained from the same parent monolayers. While electronic properties for certain nanoribbon structures converge to those of their monolayer counterpart as the two-dimensional limit is approached, other nanoribbons exhibit edge-induced states with distinct properties, such as flat band features. Our work forms the basis for designing fullerene-based nanostructures with unique advantages such as scalability and controllability.

## Results and Discussion

### From 2D to 1D

We consider two experimentally known crystalline phases of monolayer fullerene networks, i.e., the quasi-tetragonal phase (qTP) and the quasi-hexagonal phase (qHP).^20^ Figure 1a shows the crystal structures of the qTP phase. In monolayer qTP networks, neighboring carbon cages are linked by vertical [2 + 2] cycloaddition bonds along the *a*_1_ direction, while the C_60_ molecules are connected by horizontal [2 + 2] cycloaddition bonds along *a*_2_. Therefore, we denote the nanoribbons forming along the *a*_2_ direction in Figure 1b as qTP-H nanoribbons and the nanoribbons forming along *a*_2_ in Figure 1c as qTP-V nanoribbons.

**Figure 1:**
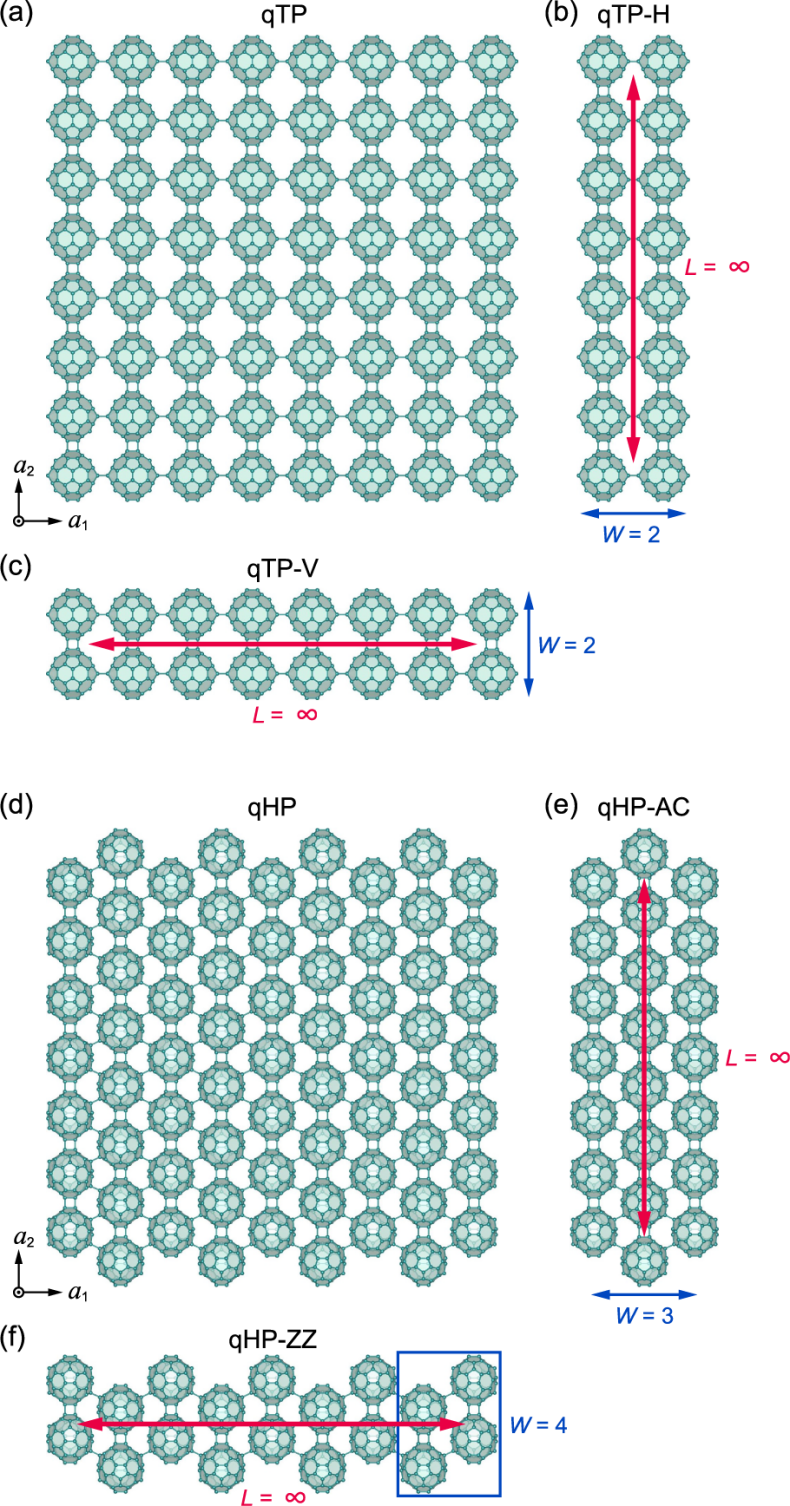
Crystal structures of (a) monolayer qTP fullerene networks, (b) qTP-H and (c) qTP-V nanoribbons, as well as (d) monolayer qHP fullerene networks, (e) qHP-AC and (f) qHP-ZZ nanoribbons.

Different from the qTP C_60_ networks, the qHP shown in Figure 1d has a closely packed structure with C–C single bonds connecting adjacent fullerene units along the diagonal of *a*_1_ and *a*_2_ and nearly horizontal [2 + 2] cycloaddition bonds connecting neighboring units along *a*_2_. Consequently, the nanoribbons forming along the *a*_2_ direction have the armchair structure in Figure 1e and we denote them as qHP-AC nanoribbons. The nanoribbons forming along the *a*_1_ direction exhibit a zigzag-like structure in Figure 1f, and we denote them as qHP-ZZ nanoribbons. Overall, there are four edge geometries: qTP-H, qTP-V, qHP-AC, and qHP-ZZ. We quantify the width of the nanoribbons, *W*, as the number of C_60_ molecules across the periodic direction. We study nanoribbon structures with *W* > 1, contrary to a purely 1D polymeric fullerene chain reported previously.^22^

### qTP-H

We first focus on qTP-H nanoribbons. Figure 2a shows the representative crystal structure of a qTP-H nanoribbon with *W* = 4. This nanoribbon has a lattice constant of 9.05 Å and a width of 34.33 Å. The space group of qTP-H nanoribbon is *Pmmm* (No. 47) with inversion symmetry, as well as *c*_2_ rotational and mirror symmetry with respect to the *x*, *y* and *z* axes.

**Figure 2:**
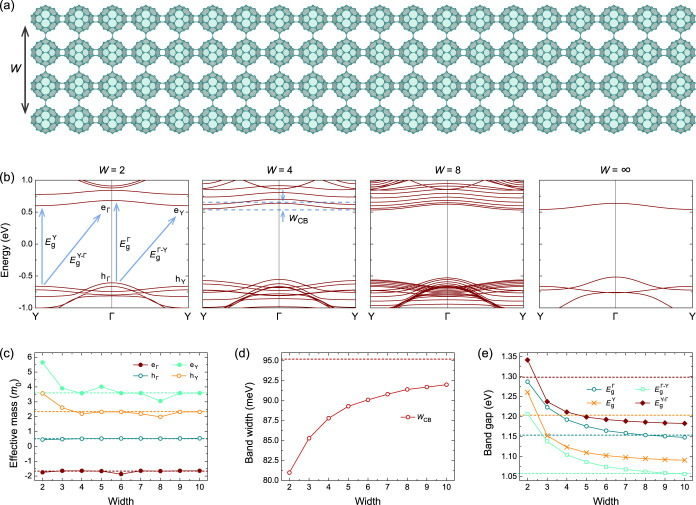
(a) Crystal structures of qTP-H nanoribbons with a representative width *W* of 4. (b) Band structures, (c) effective masses, (d) band widths, and (e) band gaps of qTP-H nanoribbons as a function of *W*. The dashed lines in (c–e) indicate the “bulk” value of the monolayer phase, corresponding to *W* = ∞.

In Figure 2b, we show the evolution of the band structures as a function of *W*, where similar features are observed by increasing *W* from 2 to ∞ (for all band structures for *W* = 1–10, see Figure S1). Increasing *W* leads to more replicas of electronic states. As an example, the two lowest conduction bands for *W* = 2 show similar curvatures, while the conduction bands with similar curvatures are doubled for *W* = 4. There are two valence band maxima (VBM) at Γ and Y, denoted as h_Γ_ and h_Y_, respectively, as well as one conduction band minimum (CBM) at Y and the negative-curvature, lowest conduction band around Γ, denoted as e_Y_ and e_Γ_, respectively.

We then investigate the evolution of effective masses of the band edges in Figure 2c. For band edges at Y, the effective masses for h_Y_ and e_Y_ decrease with *W*, until converging to the monolayer masses when *W* ≥ 9. The band edges at Y have large effective masses with *m*(h_Y_) > 2*m*_0_ and *m*(e_Y_) > 3*m*_0_, as the band edges around Y are less dispersive than those around Γ. For h_Γ_ and e_Γ_, the effective masses remain nearly unchanged and are comparable to the monolayer masses. The band edges in the vicinity of Γ have smaller effective masses as they are more dispersive, especially for h_Γ_ with effective masses around 0.5*m*_0_. Interestingly, e_Γ_ always has negative effective mass *m*(e_Γ_) ∼ −1.7*m*_0_ even for *W* = ∞. Therefore, the electron and hole at Γ have negative total mass [*m*(e) + *m*(h)] but positive reduced mass [1/*m*(e) + 1/*m*(h)]. In a classical picture, the electron–hole pairs at Γ are expected to form excitons that jointly orbit around a common center which does not lie between the two particles.^49^

While there are many crossing valence bands near the Fermi level, the lowest conduction band is relatively isolated from the other conduction bands. We can therefore define the conduction bandwidth *w*_CB_ properly. We display *w*_CB_ as a function of the nanoribbon widths *W* in Figure 2d. The smallest *w*_CB_ of 81 meV is observed for *W* = 2. The bandwidth increases monotonically with the width of the nanoribbon *W*, as the lowest conduction band becomes more dispersive, approaching that of the monolayer, 95 meV.

We next study the band gaps of qTP-H nanoribbons. As summarized in Figure 2b, there are four possible electronic transitions, depending on the point of the Brillouin zone at which they occur: the two transitions involving direct band gaps at Γ and Y, denoted as $E_{g}^{\Gamma }$ and $E_{g}^{Y}$ respectively; the transition involving indirect band gaps from h_Y_ to e_Γ_, denoted as $E_{g}^{Y-\Gamma }$ ; and the transition involving indirect band gaps from h_Γ_ to e_Y_, denoted as $E_{g}^{\Gamma -Y}$ . The smallest band gap is $E_{g}^{\Gamma -Y}$ for all *W*, which converges to the monolayer gap of 1.06 eV for *W*  > 9. The $E_{g}^{\Gamma -Y}$ of 1.29 eV for *W* = 2 is comparable to that obtained in previous calculations of the 1D fullerene chain with *W* = 1.^22^ Previous computational studies have shown that for both 1D chain and various 2D networks of C_60_, the band gap difference between unscreened hybrid functional (HF) and DFT is around 1.23 eV.^22^ Therefore, we can shift the band gaps rigidly with this correction to estimate the HF band gaps of quasi-1D nanoribbons accurately, which provide agreeable results^21–23^ with the measured band gaps.^20,25,29^ The direct band gaps at Γ also converges to the monolayer gap with increased *W*. This is unsurprising, as their corresponding band edges are in similar positions. The band gaps $E_{g}^{Y-\Gamma }$ and $E_{g}^{Y}$ for the quasi-1D nanoribbons do not converge at the monolayer gaps. This is because the VBM at Y for the nanoribbons becomes higher than the monolayer VBM at Y.

### qTP-V

Different from qTP-H, the carbon cages in the qTP-V nanoribbons are connected by vertical [2 + 2] cycloaddition bonds along the nanoribbon direction, while the cages perpendicular to the nanoribbons are linked by the horizontal cycloaddition bonds, as shown in Figure 3a. The qTP-V nanoribbons with *W* = 4 have the same space group with qTP-H but a slightly longer lattice constant of 9.13 Å and a slightly smaller width of 33.90 Å, suggesting a structural asymmetry between qTP-V and qTP-H nanoribbons.

**Figure 3:**
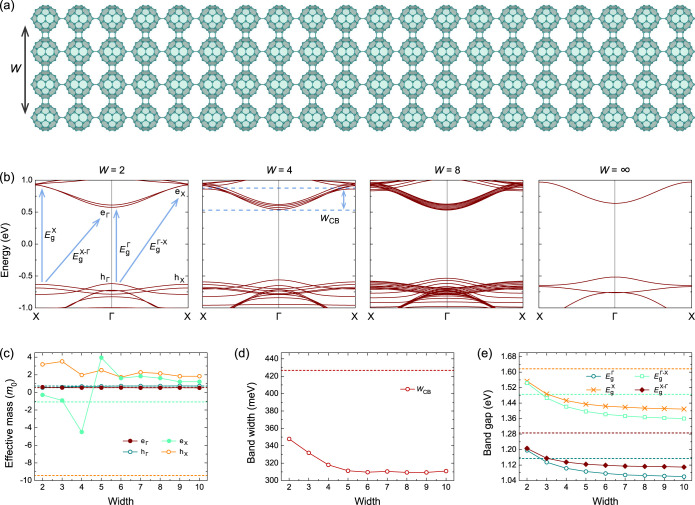
(a) Crystal structures of qTP-V nanoribbons with a representative width *W* of 4. (b) Band structures, (c) effective masses, (d) band widths, and (e) band gaps of qTP-V nanoribbons as a function of *W*. The dashed lines in (c–e) indicate the “bulk” value of the monolayer phase, corresponding to *W* = ∞.

The band structures of qTP-V nanoribbons exhibit distinct behaviors compared to qTP-H nanoribbons (for all band structures for *W* = 1–10, see Figure S2). We find direct band gap features for all *W* in Figure 3b, mainly because the e_Γ_ state has much lower energy than e_X_. With increased *W*, the electronic structure have more replicas of the bands, while the direct band gap remains stable. For e_X_ with *W* = 4, extra bands from higher conduction states become lower in energy, leading to a sudden increase of the corresponding effective masses.

Figure 3c shows the evolution of effective masses as a function of *W*. The negative *m*(e_Y_) changes its sign abruptly when *W* increases from 4 to 5 owing to the lowering of higher conduction bands as expected. This leads to a different *m*(e_Y_) from the monolayer, where the lowest conduction band is isolated from higher bands. Similarly, the h_X_ for finite *W* with positive curvature is contributed by lower valence bands as well, but the highest valence band is relatively isolated for *W* = ∞ with small negative curvature around X. For the band edges at Γ, the effective masses are nearly a constant from *W* = 2 to *W* = ∞, as the VBM and CBM at Γ of the qTP-V nanoribbons are rigid shifts of the replicas.

Despite that all the bands in qTP-V nanoribbons cross with other bands, we can still choose the relatively isolated lowest conduction band and determine its bandwidth. As shown in Figure 3d, the *w*_CB_ decreases because of the lowering of the conduction bands at X from the crossed higher conduction bands, leading to much smaller bandwidth of ∼310 meV compared to that observed in the monolayer, 427 meV.

All the band gaps of qTP-V nanoribbons reported in Figure 3e deviate significantly from the monolayer case. The smallest band gap results from the direct transition at the Γ point. At *W* = 2, the direct $E_{g}^{\Gamma }$ is only slightly smaller than the indirect $E_{g}^{X-\Gamma }$ . However, their difference becomes larger when *W* is increased, leading to more distinct direct band gap features. On the other hand, the direct band gap at X is always the largest, and the difference between the direct $E_{g}^{\Gamma }$ and the second largest indirect $E_{g}^{\Gamma -X}$ also increases for larger *W*.

### qHP-AC

Monolayer qHP networks exhibit structural features differing from qTP. The C_60_ molecules are arranged in a space-efficient manner, as shown in Figure 1d. We first focus on qHP-AC nanoribbons with the armchair-like edges, the crystal structure of which is shown in Figure 4a. The molecular cages along the nanoribbons are connected by nearly planar [2 + 2] cycloaddition bonds, while the cages across the nanoribbons are linked by intermolecular C–C single bonds. The space group of qHP-AC nanoribbons is *P*222_1_ (No. 17) with a lattice constant of 9.15 Å. A width of 30.75 Å for *W* = 4. The qHP-AC width is shorter than those in qTP nanoribbons owing to the closely packed qHP structures.

**Figure 4:**
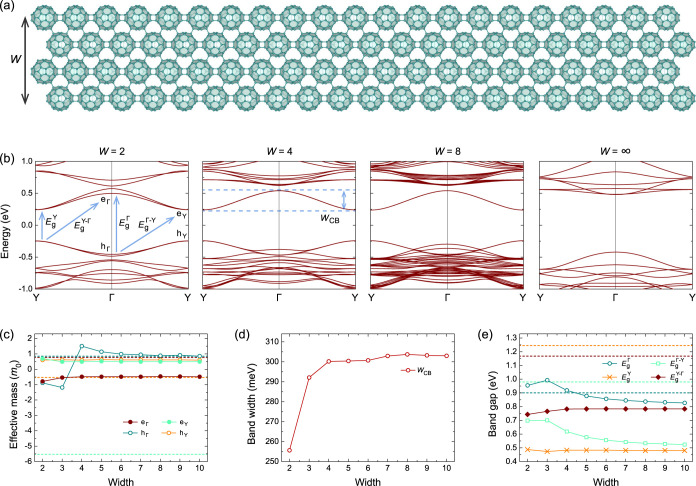
(a) Crystal structures of qHP-AC nanoribbons with a representative width *W* of 4. (b) Band structures, (c) effective masses, (d) band widths, and (e) band gaps of qHP-AC nanoribbons as a function of *W*. The dashed lines in (c,e) indicate the “bulk” value of the monolayer phase, corresponding to *W* = ∞.

The band structures of qHP-AC nanoribbons exhibit extra in-gap states compared to qHP monolayers in Figure 4b. These in-gap states are almost purely contributed by the molecules on the two edges of the nanoribbons (for all band structures for *W* = 1–10 and the corresponding fat bands, see Figure S3). While the lower valence bands and higher conduction bands show band replicas with the number of replicas proportional to *W*, the number of in-gap states is fixed for all *W*. For *W* = 2, there are two nearly degenerate in-gap valence states and two in-gap conduction states in an energy window between −0.5 and 0.5 eV. With increasing *W*, the two in-gap valence/conduction states become completely degenerate, as the interactions between the edge states is reduced when the two edges are separated.

We then consider the evolution of effective masses with increasing *W*. Because the CBM at both Γ and Y for qHP-AC nanoribbons are contributed purely by the edge states, their corresponding effective masses converge quickly to a constant value when *W* > 2. The same conclusion holds for the VBM at Y. However, the effective masses *m*(e_Γ_), *m*(e_Y_), and *m*(h_Y_) of the nanoribbons are completely different from those of the qHP monolayer. This is expected as these effective masses are from the edge states instead of the monolayer states. For h_Γ_, the monolayer states at Γ become higher than the edge states when *W*  > 3. This leads to an abrupt change of signs of the effective mass *m*(h_Γ_) from *W* = 3 to *W* = 4. Interestingly, the effective masses *m*(h_Γ_) of the qHP-AC nanoribbons start to converge to that of the monolayers when *W* > 6, as the VBM at Γ shows similar curvatures.

For qHP-AC nanoribbons, the edge states of the lowest conduction band are quite isolated from the monolayer bands. The bandwidth *w*_CB_ of 255 meV for *W* = 2 increases with larger *W*, leading to a converged bandwidth *w*_CB_ of 303 meV for *W* > 6 (Figure 4d). This also leads to a nearly constant *w*_CB_ similar to the fixed *m*(e_Γ_) and *m*(e_Y_).

Similar to the effective masses and the band widths, the band gaps of qHP-AC nanoribbons also converge to those of the edge states, as shown in Figure 4e. The direct band gap at Γ is the smallest band gap for qHP monolayers, whereas the direct band gap at Y is the smallest band gap for qHP-AC nanoribbons. The presence of edge states leads to a band gap difference of 420 meV between qHP monolayers and qHP-AC nanoribbons, which might explain the difference in the measured electronic band gaps of 1.60–2.05 eV,^20,25,29^ and optical band gaps of 1.10–1.55 eV,^20,35^ due to the finite size of the samples. As the energy of h_Γ_ becomes higher with increased *W*, the band gap $E_{g}^{\Gamma -Y}$ decreases, and the difference between $E_{g}^{\Gamma -Y}$ and $E_{g}^{Y}$ reduces.

### qHP-ZZ

Figure 5a shows the crystal structures of qHP-ZZ nanoribbons. The zigzag edge is made of the triangular edges of neighboring C_60_ connected by the diagonal single bonds. The nanoribbon width *W* is defined as the number of fullerene molecules in the primitive unit cell of the nanoribbon, as demonstrated by *W* = 8 in Figure 5a. The qHP-ZZ nanoribbons with odd *W* have a space group of *P*2/*m* (No. 10), with inversion symmetry, as well as *c*_2_ and mirror symmetry with respect to *y* axis. On the other hand, nanoribbons with even *W* have a space group of *Pma*2 (No. 28) with *c*_2_ rotational symmetry along *z* and glide mirror symmetry with respect to both *x* and *y* axis. The lattice constant for *W* = 8 is 15.85 Å, and the width of 38.96 Å is larger than the qTP-H, qTP-V, and qHP-AC nanoribbons for *W* = 4 because of the additional fullerene cage.

**Figure 5:**
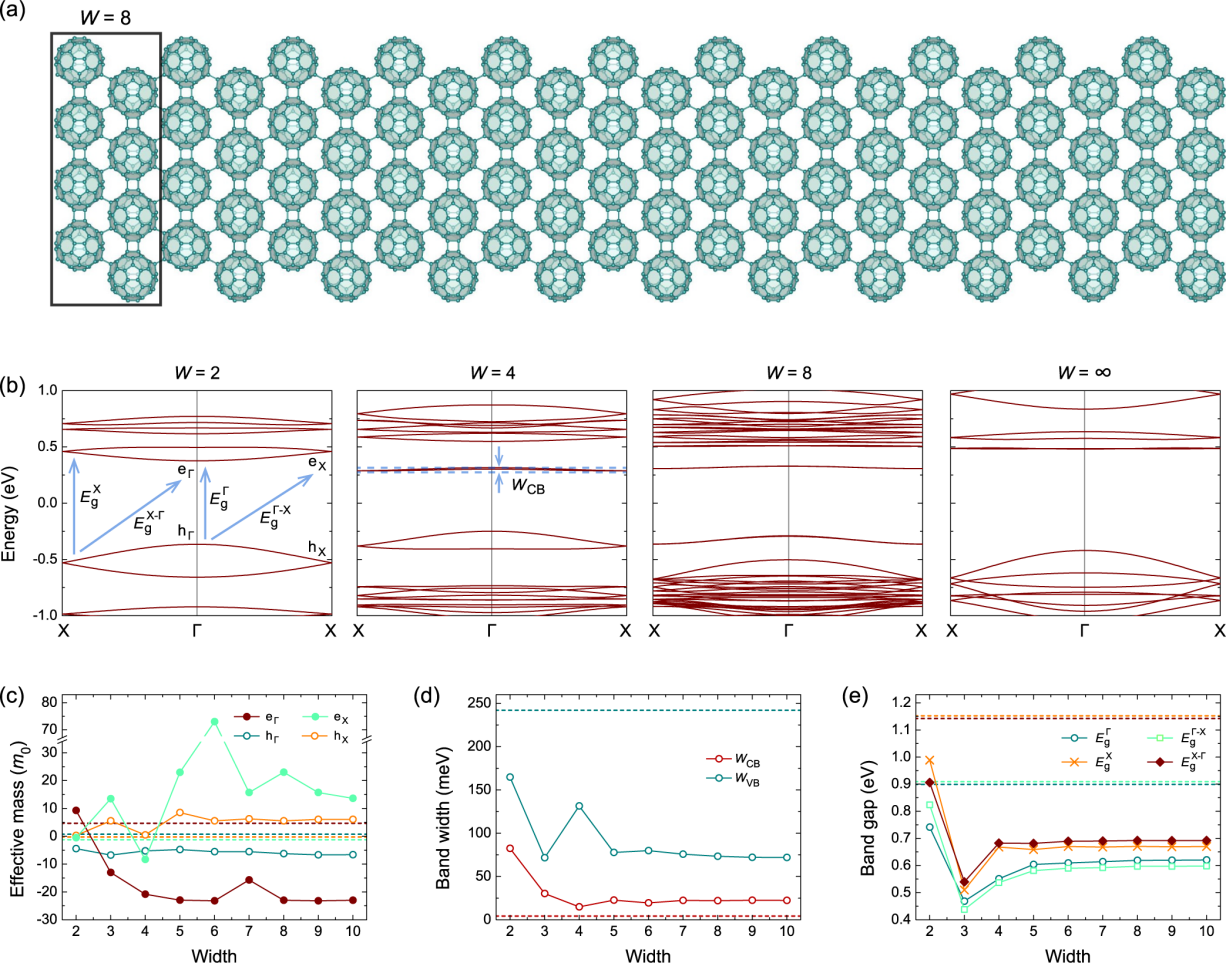
(a) Crystal structures of qHP-ZZ nanoribbons with a representative width *W* of 8. (b) Band structures, (c) effective masses, (d) band widths, and (e) band gaps of qHP-ZZ nanoribbons as a function of *W*. The dashed lines in (c–e) indicate the “bulk” value of the monolayer phase, corresponding to *W* = ∞.

Similar to the qHP-AC nanoribbons, the band structures of qHP-ZZ nanoribbons also have in-gap edge states, as shown in Figure 5b. There are two in-gap valence bands and two in-gap conduction bands (for all band structures for *W* = 1–10 and the corresponding fat bands, see Figure S4). With increasing *W*, the two conduction bands become nearly degenerate for *W* > 4, while the two valence bands become degenerate for *W*  > 7. For *W*  > 5, the band structures start to be independent of the oddness and evenness of *W*, with the monolayer features for *W* = ∞ clearly visible, as shown by the *W* = 8 panel in Figure 5b. The dominant edge contributions to the in-gap states are indicated by the local density of states in Figure S5.

Because of the in-gap states, the effective masses of the nanoribbons show distinct features from their monolayer counterpart, as shown in Figure 5c. Notably, the in-gap conduction bands are much less dispersive than the other bands, leading to large positive effective mass for e_X_ with *m*(e_X_) > 10*m*_0_ for *W* > 4, as well as large negative effective mass for e_Γ_ with *m*(e_Γ_) < −20*m*_0_ for *W* > 7. In particular, *m*(e_X_) becomes higher than 70*m*_0_ for *W* = 6, yielding a nearly flat band.

The edge states of the qHP-ZZ nanoribbons become less dispersive with increasing *W*, as demonstrated by the conduction bandwidth *w*_CB_ in Figure 5d. The conduction bandwidth converges to 72 meV for *W* > 8, while the valence bandwidth converges to 22 meV for *W* > 6. Interestingly, the *w*_CB_ of 5 meV for qHP monolayers along Γ–X is even smaller than that of the qHP-ZZ nanoribbons. One distinct feature in electronic structures of qHP-ZZ nanoribbons is the relatively isolated top valence band. Thus, we can plot the valence bandwidth *w*_VB_ in Figure 5d. The monolayer *w*_VB_ of 242 meV is much larger than that of the nanoribbons.

The evolution of the band gaps with increasing *W* in Figure 5e shows the change from a direct band gap at Γ $\left(E_{g}^{\Gamma }\right)$ to an indirect band gap $\left(E_{g}^{\Gamma -X}\right)$ when *W* increases from 2 to 3. Further increasing *W* results in the same indirect $E_{g}^{\Gamma -X}$ as the smallest band gap, while the band gap differences among $E_{g}^{\Gamma }$ , $E_{g}^{X}$ , $E_{g}^{\Gamma -X}$ , and $E_{g}^{X-\Gamma }$ are within 94 meV. Owing to the presence of the in-gap edge states, the band gap difference between the smallest monolayer gap $E_{g}^{\Gamma }$ and the converged, smallest nanoribbon gap $E_{g}^{\Gamma -X}$ is 303 meV.

## Discussion

For the qTP nanoribbons along *a*_1_ and *a*_2_ directions, the very similar crystal structures with the same space group *Pmmm* show strong directional dependence. The qTP-H nanoribbons exhibit indirect band gap features, while the qTP-V nanoribbons have direct band gap. This is unexpected given that the qTP-H and qTP-V nanoribbons for *W* = 1 have identical band structures. It should be noticed that the top valence bands of the two nanoribbons also exhibit similar features whereas the conduction bands show distinct behaviors. To understand the microscopic mechanism underlying the distinct conduction band behaviors of the two nanoribbons, we plot the fat bands for the interfullerene bonds perpendicular to the nanoribbon direction in Figure S6. For the qHP-H nanoribbons with *W* = 1, the upward conduction band from Γ to Y is dominated by the carbon atoms perpendicular to the nanoribbons that will form the vertical intermolecular bonds for *W* ≥ 2. For the qHP-V nanoribbons, the downward conduction band from Γ to X is also dominated by carbon atoms perpendicular to the nanoribbons that will contribute to the horizontal bridge bonds for *W* ≥ 2. With increasing *W*, the formation of interfullerene bonds pushes the antibonding conduction bands to higher energies. As a result, the upward conduction bands along Γ–Y become much higher for the qHP-H nanoribbons when *W* ≥ 2. On the other hand, the downward conduction bands along Γ–X shift to higher energies for the qHP-V nanoribbons when *W* ≥ 2, while the upward conduction bands along Γ–X remain nearly unchanged, which leads to the direct band gap at Γ.

The band gaps of the qHP nanoribbons exhibit strong deviations from conventional quantum confinement effects, highlighting the importance of edge effects in determining the electronic behaviors of nanostructures. Quantum confinement predicts that the band gaps of nanoribbons decreases with increasing *W*. This is observed in qTP-H and qTP-V nanoribbons (Figures 2 and 3 respectively) where the band structures are merely replicas of the “bulk” bands. However, the CBM and VBM of qHP nanoribbons are mainly consist of in-gap edge states, as shown by the fat bands in Figures S3d and S4h. The trend in the edge states exhibit strong deviations from the trend in the “bulk” states that is governed by quantum confinement. For qHP-AC nanoribbons, *W* = 2 corresponds to a width of 14.90 Å, and consequently, the interactions between the top and bottom edges are relatively weak. As a result, the smallest band gap $E_{g}^{Y}$ remains nearly a constant for *W* ≥ 2. For qHP-ZZ nanoribbons with *W* = 2, while the nanoribbon width is 11.57 Å, the distance between the top and bottom edges is only 6.98 Å, leading to more dispersive edge bands with strong splitting between the top-edge and bottom-edge bands. For *W* > 2, increasing *W* makes the interactions between the two edges weaker, which increases the band degeneracy of the top- and bottom-edge states. Further increasing *W* to 6 gives rise to nearly degenerate edge states and thus less dispersive edge bands (see Figure S4c–f). Consequently, the band gap becomes nearly a constant for *W* ≥ 6. Thus, in qHP-ZZ nanoribbons, the band gap increases with increasing *W* when *W* > 2, which is attributed to the reduced interaction strength between the top and bottom edges.

We also compare the DFT band structures with those computed from the model Hamiltonian approach^50^ based on the Wannier function^51–54^ in Figures S3k–t and S4k–t. Both the edge and “bulk” states show strong deviations between the DFT and model Hamiltonian results. The strong deviations may originate from the delocalized π electrons on the surface of C_60_,^21^ that cannot be described by the maximally localized Wannier function. This, again, highlights unexpected electronic behaviors in fullerene nanoribbons, which cannot be anticipated from theory without undertaking systematical calculations.

For experimental synthesis of different fullerene nanoribbons, all four nanoribbons have negative formation energy with respect to the C_60_ molecule for *W* > 2, as shown in Figure S8. The downhill trend of the formation energy with increasing *W* also suggests large sample sizes with high crystalline quality. The qTP nanoribbons have lower formation energy than the qHP nanoribbons, indicating that the qTP nanoribbons are thermodynamically more stable than the qHP nanoribbons. This agrees with the relative thermodynamic stability of 2D qTP and qHP monolayers, as predicted in our previous work.^46^ It is therefore expected that the qTP nanoribbons are more feasible to be synthesized experimentally.

The unique structural and electronic properties of fullerene nanoribbons open promising avenues for their integration into a range of future nanoscale devices. The direction-dependent electronic behavior can lead to nanorectifiers and flexible electronics. It is also possible to engineer topological phases,^15,16^ quantum dots,^55,56^ and magnetic edge states^57,58^ in fullerene nanoribbons for quantum electronics.^4^ Given the modular nature of these molecular nanoribbons, fullerene-based nanostructures raise intriguing intriguing possibilities for molecular arrays where each C_60_ cage acts as a discrete functional unit within a periodic framework. Future experimental synthesis and device-level integration of fullerene nanoribbons will enable a new class of fullerene-derived nanotechnologies.

## Conclusions

In summary, we systematically investigate the electronic structure of fullerene nanoribbons derived from two monolayer phases for different crystalline directions with varied widths on the basis of first-principles calculations. For qTP fullerene networks, fabricating nanoribbons along the vertical or horizontal intermolecular [2 + 2] cycloaddition bonds leads to distinct electronic properties, with direct band gaps for qTP-V nanoribbons and indirect band gaps for qTP-H nanoribbons, respectively. For qHP nanoribbons, there are extra in-gap edge states for both conduction and valence bands, and such edge states result in direct band gaps for qHP-AC nanoribbons but indirect band gaps for qHP-ZZ nanoribbons with *W* > 2. Our work reveals a rich variety of electronic properties emerging in fullerene nanoribbons depending on the details of their crystal structures, possibly laying the foundation for the design of scalable fullerene-based nanodevices.

## Methodology

Density functional theory (DFT) calculations are performed using the SIESTA package,^59–61^ under the spin-polarized, generalized-gradient approximation (GGA) of Perdew, Burke, and Ernzerhof (PBE).^62^ A double-ζ plus polarization (DZP) basis set is used with an energy cutoff of 400 Ry and a reciprocal space sampling of 18 *k*-points along the periodic direction. A vacuum spacing in the nonperiodic directions larger than 20 Å is used throughout. Both the lattice constant and atomic positions are fully relaxed using the conjugate gradient method,^63^ with a tolerance on forces of 0.02 eV/Å. For monolayer qTP and qHP networks, the inclusion of the Grimme’s D3 dispersion corrections,^64^ leads to a decrease in lattice constants by merely 0.3%, which is consistent with our previous calculations.^21,23^ We therefore neglect the van der Waals interactions hereafter. For electronic structures, we use 100 *k*-points to sample the high-symmetry line. The effective mass is defined as 1/*m* = (1/ℏ^2^)­(∂^2^ *E*/∂*k* ^2^) and computed by quadratic fitting of eigenenergies of six *k*-points near the band edge. For relatively isolated bands, the bandwidth is defined by the difference between the highest and lowest eigenenergies. The indirect band gap $E_{g}^{A-B}$ represents the eigenenergy difference between conduction band minimum (electrons, denoted as “e”) at high-symmetry point B and the valence band maximum (holes, denoted as “h”) at high-symmetry point A, i.e., $E_{g}^{A-B}=E\left(e_{B}\right)-E\left(h_{A}\right)$ .

## Supplementary Material



## Notes

Electronic structures of qTP-H fullerene nanoribbons for *W* from 1 to 10 (Figure S1); electronic structures of qTP-V fullerene nanoribbons for *W* from 1 to 10 (Figure S2); electronic structures of qHP-AC fullerene nanoribbons for *W* from 1 to 10 based on SIESTA and WannierTools (Figure S3); electronic structures of qHP-ZZ fullerene nanoribbons for *W* from 1 to 10 based on SIESTA and from 2 to 20 with a step of 2 based on WannierTools (Figure S4); local density of states for C_60_ molecules on the edge and in the “bulk” (Figure S5); fat bands for qTP-H and qTP-V nanoribbons with *W* = 1 (Figure S6); PBE0 and PBE band gap for different phases of 0D, 1D, 2D, and 3D C_60_ (Figure S7); formation energy of fullerene nanoribbons with respect to C_60_ molecules (Figure S8); energy levels of one isolated C_60_ molecule (Figure S9) (PDF)
