# Supplementary material for: Electronic Structure of Fullerene Nanoribbons
Source: ACS Nano. 2025 Jul 30;19(32):29637–45. doi: 10.1021/acsnano.5c08991 (PMC12368996; doi:10.1021/acsnano.5c08991)
Supplement: Supplementary file 1 [file nn5c08991_si_001.pdf]

Supporting Information for "Electronic structure of fullerene nanoribbons"

Bo Peng*,† and Michele Pizzochero*,‡,¶

† *Theory of Condensed Matter Group, Cavendish Laboratory, University of Cambridge, Cambridge CB3 0HE, United Kingdom*

‡ *Department of Physics, University of Bath, Bath BA2 7AY, United Kingdom*

¶ *School of Engineering and Applied Sciences, Harvard University, Cambridge, Massachusetts 02138, United States*

E-mail: bp432@cam.ac.uk; mp2834@bath.ac.uk

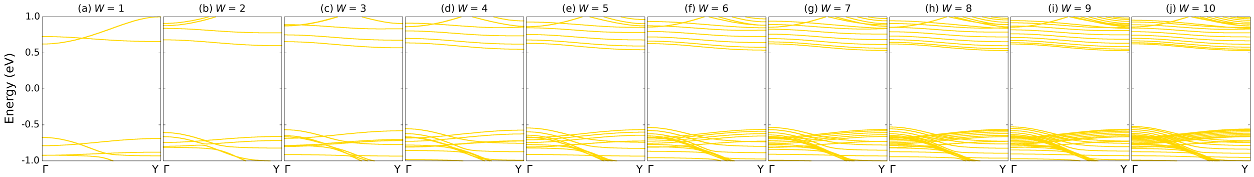

Figure S1: (a)-(j) Electronic structures of qTP-H fullerene nanoribbons for W from 1 to 10.

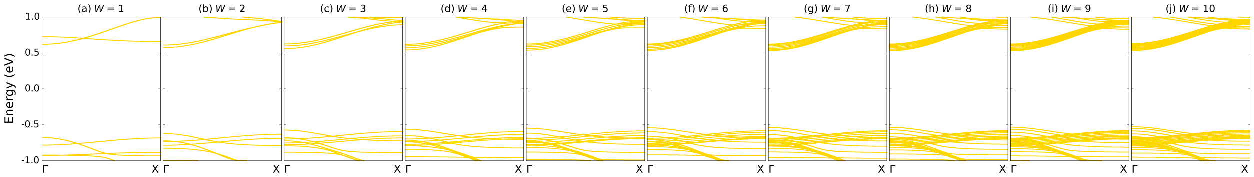

Figure S2: (a)-(j) Electronic structures of qTP-V fullerene nanoribbons for W from 1 to 10.

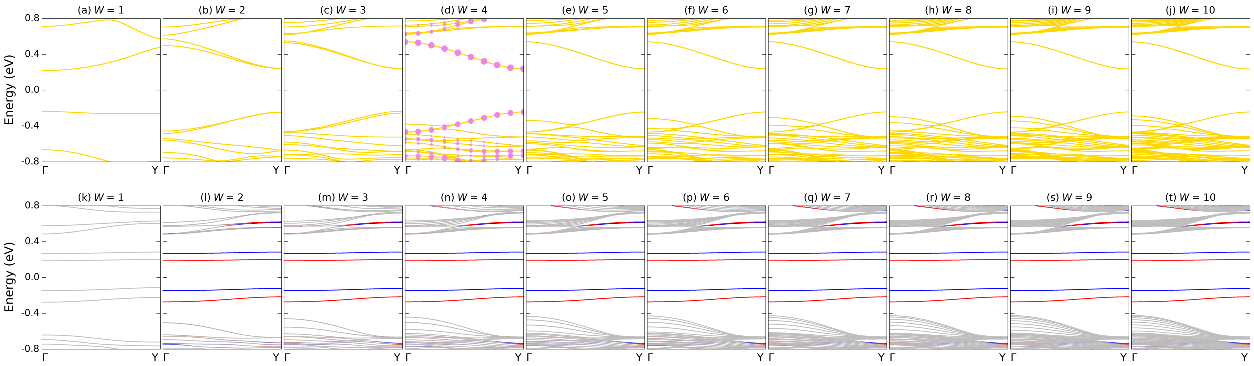

Figure S3: Electronic structures of qHP-AC fullerene nanoribbons for W from 1 to 10 based on (a)-(j) SIESTA [1–3] and (k)-(t) WannierTools.[4] The fat bands for $W = 4$ in (d) indicate contributions from the C_{60} molecules on both edges. The red/blue color in (k)-(t) indicates contributions from the top/bottom edges.

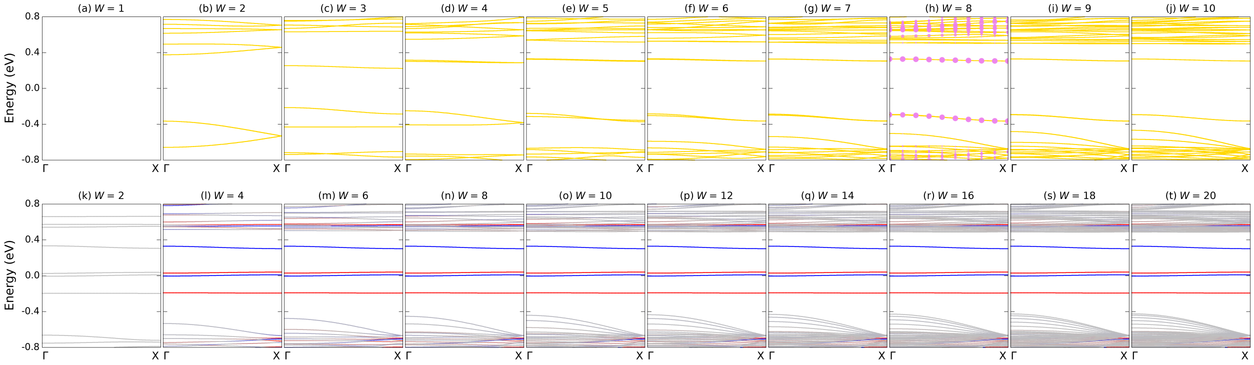

Figure S4: Electronic structures of qHP-ZZ fullerene nanoribbons for W (a)-(j) from 1 to 10 based on SIESTA [1–3] and (k)-(t) from 2 to 20 with a step of 2 based on WannierTools.[4] The fat bands for $W = 8$ in (h) indicate contributions from the C_{60} molecules on both edges. The red/blue color in (k)-(t) indicates contributions from the top/bottom edges.

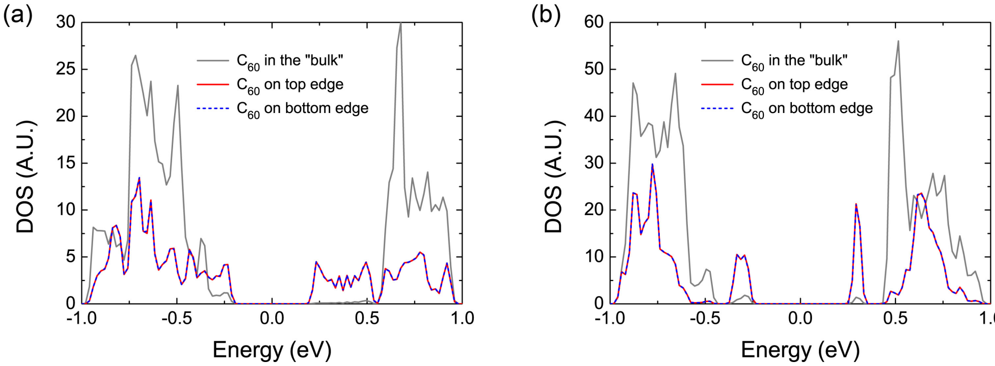

Figure S5: Local density of states for C_{60} molecules on the edge and in the "bulk" for (a) qHP-AC nanoribbons with $W = 4$ and (b) qHP-ZZ nanoribbons with $W = 8$, indicating that the in-gap bands are mainly consist of the edge states.

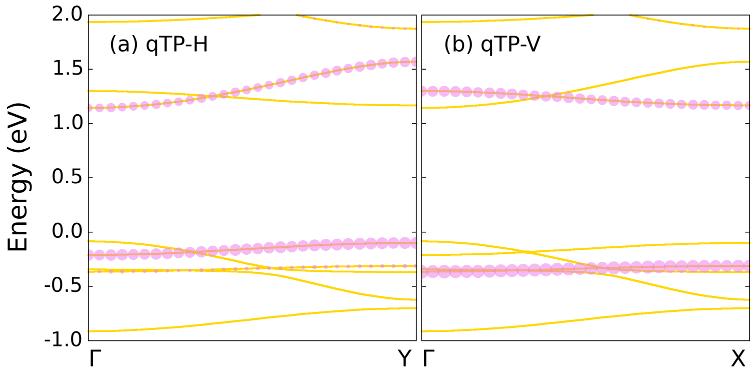

Figure S6: (a) Fat bands for qTP-H nanoribbons with $W = 1$. The purple circles indicate contributions from the carbon atoms in the vertical intermolecular bonds perpendicular to the nanoribbon direction. (b) Fat bands for qTP-V nanoribbons with $W = 1$. The purple circles indicate contributions from the carbon atoms in the horizontal intermolecular bonds perpendicular to the nanoribbon direction.

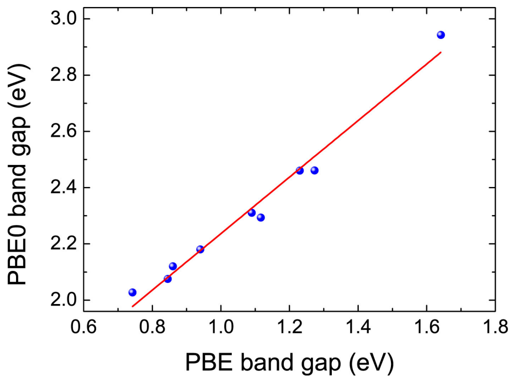

Figure S7: PBE0 and PBE band gap for different phases of 0D, 1D, 2D, and 3D C_{60}.

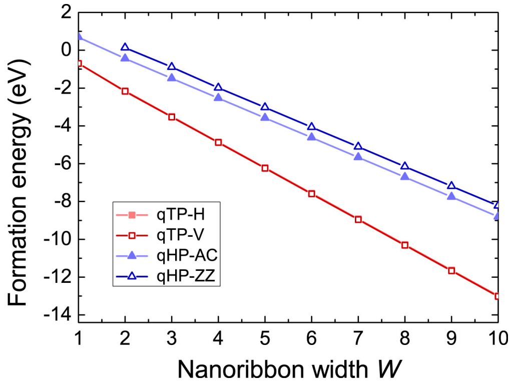

Figure S8: Formation energy of fullerene nanoribbons with respect to C_{60} molecules.

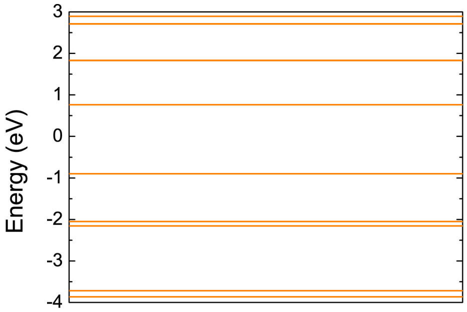

Figure S9: Energy levels of one isolated C_{60} molecule.

References

(1) Soler, J. M.; Artacho, E.; Gale, J. D.; García, A.; Junquera, J.; Ordejón, P.; Sánchez-Portal, D. The SIESTA method for ab initio order-N materials simulation. J. Phys.: Condens. Matter **2002**, 14, 2745–2779.

(2) Artacho, E.; Anglada, E.; Diéguez, O.; Gale, J. D.; García, A.; Junquera, J.; Martin, R. M.; Ordejón, P.; Pruneda, J. M.; Sánchez-Portal, D.; Soler, J. M. The SIESTA method; developments and applicability. J. Phys.: Condens. Matter **2008**, 20, 064208.

(3) García, A. et al. Siesta: Recent developments and applications. J. Chem. Phys. **2020**, 152, 204108.

(4) Wu, Q.; Zhang, S.; Song, H.-F.; Troyer, M.; Soluyanov, A. A. WannierTools: An open-source software package for novel topological materials. Computer Physics Communications **2018**, 224, 405–416.
